# Reducing overdiagnosis by polygenic risk-stratified screening: findings from the Finnish section of the ERSPC

**DOI:** 10.1038/bjc.2015.289

**Published:** 2015-08-20

**Authors:** Nora Pashayan, Paul DP Pharoah, Johanna Schleutker, Kirsi Talala, Teuvo LJ Tammela, Liisa Määttänen, Patricia Harrington, Jonathan Tyrer, Rosalind Eeles, Stephen W Duffy, Anssi Auvinen

**Affiliations:** 1 Department of Applied Health Research, University College London, 1-19 Torrington Place, London WC1E 7HB, UK; 2 Centre for Cancer Genetic Epidemiology, Department of Public Health and Primary Care, University of Cambridge, Strangeways Research Laboratory, Worts Causeway, Cambridge CB1 8RN, UK; 3 Department of Medical Biochemistry and Genetics, University of Turku, Turku FI20014, Finland; 4 Finnish Cancer Registry, Helsinki FI 00130, Finland; 5 Department of Surgery, Tampere University Hospital and School of Medicine, University of Tampere, Tampere FI 33014, Finland; 6 Division of Genetics and Epidemiology, The Institute of Cancer Research & Royal Marsden NHS Foundation Trust, London SM2 5NG, UK; 7 Centre for Cancer Prevention, Mathematics and Statistics, Wolfson Institute of Preventive Medicine, Queen Mary University of London, Charterhouse Square, London EC1M 6BQ, UK; 8 School of Health Sciences, University of Tampere, Tampere FI 33014, Finland

**Keywords:** ERSPC-Finland, overdiagnosis, polygenic risk, prostate cancer, stratified screening

## Abstract

**Background:**

We derived estimates of overdiagnosis by polygenic risk groups and examined whether polygenic risk-stratified screening for prostate cancer reduces overdiagnosis.

**Methods:**

We calculated the polygenic risk score based on genotypes of 66 known prostate cancer loci for 4967 men from the Finnish section of the European Randomised Study of Screening for Prostate Cancer. We stratified the 72 072 men in the trial into those with polygenic risk below and above the median. Using a maximum likelihood method based on interval cancers, we estimated the mean sojourn time (MST) and episode sensitivity. For each polygenic risk group, we estimated the proportion of screen-detected cancers that are likely to be overdiagnosed from the difference between the observed and expected number of screen-detected cancers.

**Results:**

Of the prostate cancers, 74% occurred among men with polygenic risk above population median. The sensitivity was 0.55 (95% confidence interval (CI) 0.45–0.65) and MST 6.3 (95% CI 4.2–8.3) years. The overall overdiagnosis was 42% (95% CI 37–52) of the screen-detected cancers, with 58% (95% CI 54–65) in men with the lower and 37% (95% CI 31–47) in those with higher polygenic risk.

**Conclusion:**

Targeting screening to men at higher polygenic risk could reduce the proportion of cancers overdiagnosed.

At 13 years of follow-up, the European Randomised Study of Screening for Prostate Cancer (ERSPC) showed a 21% relative reduction in prostate cancer mortality in favour of screening, with one prostate cancer death prevented and 27 additional cases detected per 781 men invited to screening (Schroder *et al*, 2014). The number needed to detect and the number needed to invite to prevent one prostate cancer death were less at 13 years than at 9 and 11 years of follow-up. Despite the improvement in the absolute benefit of screening, concerns about the negative consequences of screening, mainly overdiagnosis and treatment of overdiagnosed cancers, remain obstacles for large-scale screening. An overdiagnosed cancer is defined as one that would not have presented clinically in a person's lifetime in the absence of screening. Developing methods to reduce overdiagnosis remains crucial for diminishing the adverse effects of screening.

To date, genome-wide association studies have identified 100 prostate cancer susceptibility loci, which explain ∼33% of the familial risk of prostate cancer in population of European ancestry (Al Olama *et al*, 2014). Assuming a log-additive model of interaction between loci, the currently known loci define a polygenic risk profile that could be used for risk stratification (Pharoah *et al*, 2008; Pashayan *et al*, 2011). Men at 90th and 99th percentile of the risk distribution are at 2.9- and 5.7-fold increased risk for prostate cancer compared with the average population (Al Olama *et al*, 2014).

A personalised screening strategy based on age and genetic risk has the potential to improve the efficiency of the screening programme (Pashayan *et al*, 2011) and reduce its adverse consequences (Pashayan *et al*, 2015). A mathematical modelling study using UK-based prevalent screen and incident cancer data has shown that the proportion of screen-detected cancers likely to be overdiagnosed varies inversely by polygenic risk (Pashayan *et al*, 2015). However, the estimates of overdiagnosis were based on mean sojourn time (MST) and test sensitivity derived from the published literature. Using screening trial data from the Finnish section of ERSPC, this study aims to estimate MST and sensitivity and then use these estimates to derive the probability of overdiagnosis by polygenic risk. This is to examine whether a risk-stratified screening strategy reduces the proportion of cancers overdiagnosed.

## Materials and Methods

### Study participants

**Figure 1:**
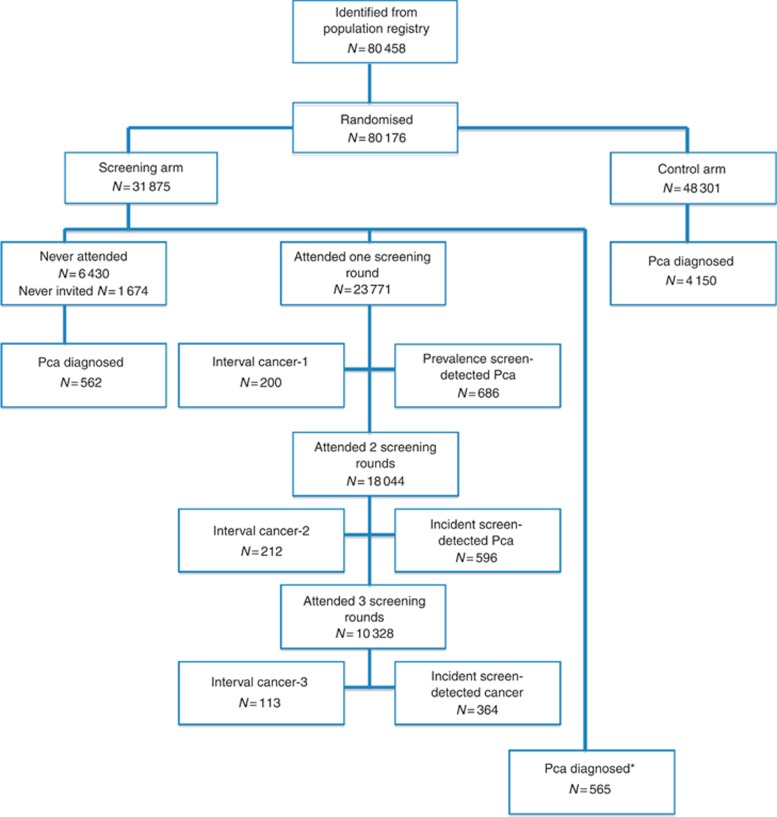
**Flow chart of the Finnish Prostate Cancer Screening Trial.** *Cancer diagnosed more than 4 years since the last screen; 282 men were excluded from randomisation because of death, or prostate cancer diagnosis. Pca, prostate cancer.

The Finnish Prostate Cancer Screening Trial is the largest component of the ERSPC. The trial population and the protocol have been described in detail elsewhere (Finne *et al*, 2003). Briefly, during 1996 and 1999, a total of 80 458 men aged 55, 59, 63, and 67 years were identified from the Finnish Population Registry and of whom 80 176 were randomised to either the screening arm (*N*=31 875) or the control arm (*N*=48 301). Men in the screening arm were invited for serum prostate specific antigen (PSA) testing every 4 years up to three times until age 71 years. The study protocol was approved by the ethical committees of Helsinki University Hospital and Tampere University Hospital. Figure 1 presents the number of men in the trial and cancers detected.

Men were referred for transrectal ultrasound-guided biopsy if the PSA value was ⩾4.0 ng ml^−1^ or the PSA was 3.0–3.99 ng ml^−1^ with suspicious findings on digital rectal examination (in 1996–1998) or with free/total PSA ratio <0.16 (since 1999). Initially, sextant biopsies were used, and from 2002, 10 to 12 biopsy cores were taken.

Incident cancers diagnosed among the controls and the non-participants, and interval cancers were identified through record linkage to the nationwide population-based Finnish Cancer Registry, which has almost complete coverage of all solid cancers diagnosed in Finland (Teppo *et al*, 1994). An interval cancer was defined as cancer diagnosed in the interval 1-4 years after screening attendance. Cancers in non-participants and in those diagnosed more than 4 years after the previous screen were not regarded as interval cancers. Cancers that were not diagnosed through organised screening were referred to as clinically diagnosed.

Prostate cancer was classified as localised disease with tumour-node-metastasis (TNM) stage T1-2 N0 M0; and advanced disease (regional-distant) with stage T3 and above or any T N1 M1; as non-aggressive tumour, Gleason score <7, and aggressive tumour, Gleason score ⩾7.

The follow-up for this analysis was until 31 December 2011 and the median duration of follow-up was 13 years.

### Genotyping and quality control

At the time of genotyping, there were 70 prostate cancer susceptibility variants identified through genome-wide association studies. The analysis was based on 66 of these variants (Supplementary Table 1S) for a sample of the trial participants, 1089 men with prostate cancer and 3878 men without prostate cancer. Genotyping platform (Wang *et al*, 2009; Eeles *et al*, 2013) and quality control are described in the Supplementary File.

### Polygenic risk score and absolute risk calculations

A polygenic risk score (PRS) for each individual was calculated as:



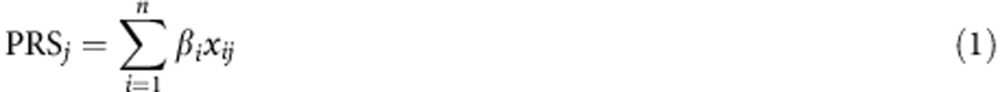



where *β*_*i*_ is the per-allele log-odds ratio for locus *i*, *x*_*ij*_ represents the number of risk alleles (i.e., 0, 1 or 2) carried by each individual *j* at locus *i*, and *n* is the number of loci.

The risk conferred by each of the variants is assumed to be allele dose-dependent with a multiplicative (log-additive) effect on a relative-risk scale (Pharoah *et al*, 2002). Under the multiplicative model the distribution of polygenic risk in the population at birth follows the normal distribution when relative risk is plotted on a logarithmic scale, with mean, *μ*, and variance *σ*^2^. We set the mean, *μ* =−*σ*^2^/2, so that the mean relative risk in the population at birth is equal to unity. The distribution of relative risk among cases at young ages is also log-normal with the same variance, but with larger mean, *μ* + *σ*^2^ (Pharoah *et al*, 2002).

### Estimating overdiagnosis

#### Mean sojourn time and sensitivity of PSA

Assuming exponential distribution of sojourn time, we estimated the sensitivity and the MST by the maximum likelihood method of Walter and Day (Walter and Day, 1983). The likelihood was evaluated over a two-dimensional grid of values of sensitivity, *S*, and of inverse of MST, *λ*. The observed incidence of interval cancers was assumed to follow a Poisson process with an expected incidence of interval cancer that depends on *S* and *λ*, as such:



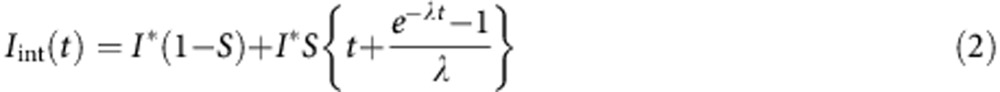



where *I*_int_*(t)* is the expected incidence rate of interval cancers at time *t*, and I* is the underlying incidence rate of prostate cancer in the absence of screening, derived from the observed incidence rate in the control arm. Here, person-years at risk were calculated from time of randomisation to time of prostate cancer diagnosis, death or censoring date (31 December 2011), whichever occurred first.

To calculate the 95% confidence level (CI), we identified the combinations of values of *S* and *λ* for which the log likelihood was 1.92 less than the maximum value (Day and Walter, 1984).

As the maximum likelihood approach is based on interval cancers, the derived estimates of *S* and *λ* refer to non-overdiagnosed cancers. Also, given how interval cancers were defined in this study, the sensitivity refers to episode sensitivity, that is, performance of the test and the diagnostic work up (Hakama *et al*, 2007).

#### Expected number of non-overdiagnosed screen-detected cancers

Given *S* and *λ*, we estimated the expected prevalence and incidence of non-overdiagnosed cancers at first and subsequent screens, as such:

Expected prevalence:



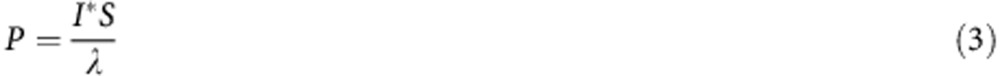



Expected incidence at second screen:



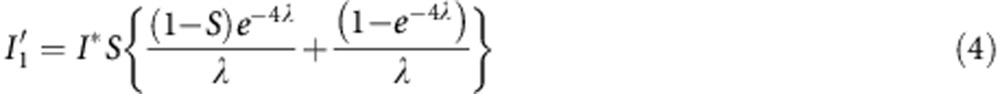



Expected incidence at third screen:



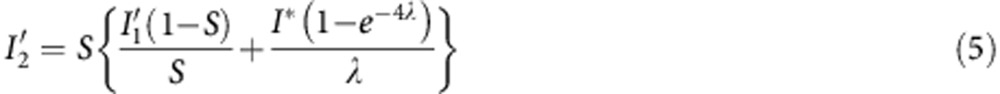



We applied the expected prevalence and incidence rate to the number of men screened at each round, and estimated the expected number of non-overdiagnosed cancers. If *O* is the observed number of screen-detected cancers, and *E* is the expected number of non-overdiagnosed cancers, then the proportion of screen-detected cancers likely to be overdiagnosed would be 
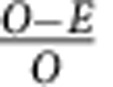
.

#### Estimation of overdiagnosis by PRS

The PRS was available on subsample of men with screen-detected cancers, interval cancers, and incident cancers, and on subsample of men without cancer. We stratified men with and without prostate cancer into two risk groups: below and above 50th percentile of polygenic risk distribution among the population, hereafter referred to as lower and higher risk groups, respectively. In the subsamples, the proportions in the higher risk groups are shown in Table 1. We used these proportions as sampling fractions to derive the likely proportion of the study population with and without prostate cancer in the lower and higher risk groups. To estimate the baseline incidence rate of prostate cancer by polygenic risk groups, we derived the relative rate of clinical cancers in the two risk groups using information on interval cancer as such:



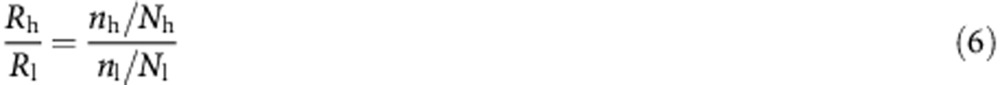



where *n*_h_ and *n*_l_ are the number of interval cancers in the higher and lower risk groups, respectively; and *N*_h_ and *N*_l_ are the number of men screened in the higher and lower risk groups, respectively.

Then the overall rate would be:



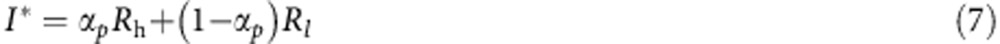



where *α*_*p*_is the sampling fraction for men free of cancer in the screening arm and in the higher risk group.

We derived separate estimates of overdiagnosis for those in the lower and higher risk groups by repeating the steps used to estimate the overall overdiagnosis.

#### Sensitivity analysis

In the Finnish Prostate Cancer Screening Trial, 19% of men in the control group had PSA test in the first 4 years. In a sensitivity analysis, we estimated the baseline incidence rate after excluding men with cancer detected following PSA testing, and re-estimated overdiagnosis by polygenic risk groups.

## Results

The distribution of PRS based on 66 prostate cancer susceptibility loci had mean (scaled mean) of −0.16 (−0.20) and variance of 0.40 among men with no prostate cancer and mean of 0.30 (0.20) and variance of 0.40 among men with the cancer. There was no statistically significant difference in the mean PRS of men with screen-detected *vs* clinically diagnosed cancer (*t*-test *P*=0.137) (Table 2). The polygenic risk scores at the 25th, 50th, and 75th percentiles of the risk distribution among men with no prostate cancer accounted for 17%, 26%, and 48% of the cases, respectively. Thus, men in the high-risk group (above the 50th centile) accounted for 74% of the cases.

The PRS was available on 35% of men with screen-detected cancer and 9% of men with cancer in the control arm. The proportions of men with screen-detected cancer with advanced stage, Gleason score ⩾7, or PSA ⩾4 were comparable between the trial participants overall and the subsample of men with PRS. However, out of clinically diagnosed cancers, the subsample with PRS had lower proportion of cancers with advanced stage or Gleason score ⩾7 compared with all clinically diagnosed cancers (Table 3).

PSA level varied by polygenic risk, 18% of men in the higher polygenic risk group compared with 7% of men in the lower risk group had PSA ≥4 ng ml^−1^ (*P*<0.001). However, after adjusting for stage and Gleason score, there was no statistically significant association between polygenic risk group and PSA categories (odds ratio=1.35, 95% CI 0.89-2.04). Among the screen-detected cancers, there was statistically significant association between polygenic risk group and stage (*P*=0.046) and Gleason score categories (*P*=0.005). However, similar association was not seen among the clinically diagnosed cancers (Table 3).

Overall, higher polygenic risk was associated with significantly increased odds of Gleason score ⩾7 tumours (odds ratio=1.56, 95% CI 1.15–2.10) but not with advanced stage (odds ratio=1.36, 95% CI 0.85-2.16).

The baseline incidence rate of prostate cancer in the control group was 6.17 cases per 1000 person-years (4150 cases/672 610 person-years from time of randomisation to censoring date). The estimated baseline incidence rates of prostate cancer were 2.47 and 9.90 cases per 1000 person-years in the lower and higher risk groups, respectively. The likelihood for the expected incidence rate of interval cancers was maximised for sensitivity of 0.55 (95% CI 0.45-0.65) and *λ* of 0.16 (95% CI 0.12-0.24) (Table 4).

Given these parameters, the expected number of screen-detected non-overdiagnosed cancers after three rounds of screening would be 950. As such 42% (95% CI 37-52; 696 out of 1646) of the observed screen-detected cancers would likely be overdiagnosed (Table 5).

The lower risk group would account for 50% of the screening episodes (*N*=26 186) in the trial and almost 26% of the observed screen-detected cancers (*N*=453). A baseline incidence of 0.00247, *S* of 0.55 and *λ* of 0.16, the expected number of non-overdiagnosed cancers would be 191. Hence, 58% (95% CI 54-65; 262 out of 453) of the screen-detected cancers in the lower PRS group would likely be overdiagnosed.

The higher risk group would account for almost 50% of the screening episodes (*N*=25 957) in the trial and almost 74% of the observed screen-detected cancers (*N*=1193). Using baseline incidence rate of 0.00990, *S* of 0.55 and *λ* of 0.16, the expected number of non-overdiagnosed cancers would be 756. Correspondingly, 37% (95% CI 31-47; 437 out of 1193) of the screen-detected cancers would likely be overdiagnosed.

At 13 years of follow-up, the overall overdiagnosed cases per 1000 men screened were 29, with 22 and 37 in the lower and higher polygenic risk groups, respectively.

Targeting screening to men in the higher risk group would miss 191 non-overdiagnosed cancers, while avoiding 262 overdiagnosed cancers. Targeted screening would reduce the overall cases overdiagnosed in the population from 29 (696 out of 23 771) to 18 (437 out of 23 771) per 1000 men.

In a sensitivity analysis, after excluding 9129 from the control group who had PSA test in the first 4 years and the expected subsequent 946 cancer diagnoses, the baseline incidence rate of prostate cancer in the control group was 5.87 cases per 1000 person-years (3204 cases/545 148 person-years from time of randomisation to censoring date). With baseline incidence rate of 0.00587, the *S* and *λ* were derived as 0.52 and 0.16, respectively. With these parameters, overall overdiagnosis was estimated as 47%, with 62% in the lower risk group and 37% in the higher risk group.

## Discussion

This study, based on the Finnish prostate cancer screening trial data, suggests that the proportion of screen-detected cancers that are likely to be overdiagnosed is inversely related to polygenic risk, that is, proportion overdiagnosed decreases with increase in polygenic risk. The proportion of screen-detected cancers that are likely to be overdiagnosed is estimated to be 37% lower in men with polygenic risk higher than the average population risk than in men with lower polygenic risk. In the Finnish population-based screening trial with three rounds of screening, 31 700 screening episodes would detect 1000 cancers in these men, of which 577 would likely be non-overdiagnosed and 423 overdiagnosed. A polygenic risk-stratified screening programme would involve polygenic profiling of all men for risk stratification. Then the screening test, the PSA, would be offered to the strata of men above a certain polygenic risk threshold. As such a subset of men are offered PSA screening. Targeting screening to men in the higher polygenic risk group is estimated to reduce screening episodes by half while detecting 80% of the non-overdiagnosed cancers and reducing overdiagnosed cancers by 38% at a cost of missing 20% of the non-overdiagnosed cancers. That is, for every non-overdiagnosed cancer not detected through screening, almost two (37/20) overdiagnosed cases could be avoided.

We have reported similar inverse association between quartiles of polygenic risk and overdiagnosis using different analysis approach and using data from the UK on prevalence screening and incident cancers only (Pashayan *et al*, 2015). However, the study was limited by taking MST and test sensitivity values from different sources. In this study, having randomised screening trial data with information on interval cancers, we estimated simultaneously the MST and episode sensitivity for non-overdiagnosed cancers and from them derived the probability of overdiagnosis in the Finnish trial setting. This enhances the validity of our results. We do acknowledge, however, that the present results rely on a number of assumptions, and that they remain subject to considerable uncertainty. There is a need for continued development of rigorous methods of estimation of overdiagnosis, including reliable confidence interval estimation and for further data on screened and unscreened populations with polygenic risk measured.

Although the proportion of screen-detected cancers that are likely to be overdiagnosed decreased with polygenic risk, the absolute rate of overdiagnosis increased with polygenic risk. This is because majority of the cancers (74%) occurred in the higher risk group. Although screening was estimated to result in 67% more overdiagnosed cancers in the higher compared with the lower risk group (437 *vs* 262), it also resulted in almost 300% more non-overdiagnosed cancers in the higher risk group (756 *vs* 191). Thus, overdiagnosis in the higher risk group is estimated to be substantially smaller as a proportion of screen-detected cancers, and would be expected to be correspondingly smaller in proportion to prostate cancer deaths avoided.

We have used the maximum likelihood method to estimate MST of 6.2 years and episode sensitivity of 55%. All estimates of MST and sensitivity are subject to both sampling variation and uncertainty due to other sources such as the distributional assumptions involved. Our estimates are within the 95% CI of previously reported estimates. Wu *et al* (2012) using multistate modelling with the same Finnish screening trial data have reported MST of 7.7 (95% CI 6.0-10.7) years and episode sensitivity of 43% (95% CI 35-51) for the first screening round and 60% (95% CI 48-72) for the second round. Our estimate of overdiagnosis of 42% is in line with the estimates from the ERSPC (Draisma *et al*, 2003). Our analysis indicates that 2.9% of men screened with three rounds of screen are likely to be overdiagnosed. This estimate is comparable to that of Wu *et al* (2012) of 3.4% (95% CI 2.1-5.7). These figures are also consistent with other studies (Etzioni *et al*, 2002; Telesca *et al*, 2008; Draisma *et al*, 2009; Loeb *et al*, 2014).

It is of interest to know whether the natural history of the cancer varies by genetic risk. However, the relatively small number of cases, particularly in the lower polygenic risk group, limited precision of the sensitivity and MST estimates by polygenic risk group. As the majority of the cancers were in the higher polygenic risk group, then the estimated MST and sensitivity are likely to reflect primarily those of that population. Preliminary analysis suggests similar episode sensitivity and longer MST in the lower polygenic risk group. MST varying by polygenic risk is plausible given the observed association between Gleason score and PRS. With longer sojourn time, we would expect higher overdiagnosis (Draisma *et al*, 2009) in the lower risk group. Risk groups with longer MST may be offered less-frequent screening. As such studying variation of MST with PRS is important for designing risk-tailored screening.

The subsample of men with genotyping data and clinically diagnosed cancer had less-aggressive and less-advanced cancers than the remaining participants diagnosed clinically. Less-aggressive cancers were associated with lower PRS. If our subsample had more aggressive cancers, then the proportion of interval cancers and baseline incidence rate in the higher risk group would have been larger, resulting in even lower estimate of overdiagnosis in the higher risk group.

Also, a sensitivity analysis accounting for some of the effect of contamination yielded almost similar results.

In this study, we have used only polygenic risk profile for stratification. Further research is needed to study the benefits, the harms, and cost-effectiveness of stratifying the population into several risk strata based on polygenic risk combined with other risk factors, such as age, family history, and baseline PSA (Loeb, 2012; Roobol and Carlsson, 2013), and offering screening differentially (different starting age, inter-screening interval, and screening modality) to each population stratum.

In summary, polygenic risk-stratified screening for prostate cancer could reduce the proportion of cancers overdiagnosed. Targeting screening to men at higher polygenic risk could improve the benefit to harm balance of screening.

## Figures and Tables

**Table 1:** Proportion of the study population with PRS and of those in the higher risk group

	**No. (%) with PRS**	**No. (%) with PRS in the higher risk group**
**Screening arm**		
Men with no prostate cancer *N*=21 030	3877 (18.4)	1930 (49.8)
Screen-detected cancer at first round of screening *N*=686	173 (25.2)	131 (75.7)
Screen-detected cancer at subsequent rounds of screening *N*=960	406 (42.3)	285 (70.2)
Interval cancer *N*=525	115 (21.9)	92 (80.0)
Clinically diagnosed cancer >4 years after last screen *N*=565	34 (6.0)	25 (73.5)
Non-participants with clinically diagnosed cancer *N*=562	22 (3.9)	17 (77.3)
Non-participants with no cancer diagnosis *N*=7542	0 (0.0)	0 (0.0)
**Control arm**		
Clinically diagnosed cancer *N*=4150	339 (8.2)	250 (73.8)
No cancer *N*=44 151	1 (0.0)	1 (100.0)

Abbreviation: PRS=polygenic risk score.

**Table 2:** Polygenic risk distribution among men with and without cancer in the Finnish screening trial

	**Mean**	**Scaled mean** a	**Variance**
Men without prostate cancer *N*=3878	−0.160	−0.198	0.397
Men with prostate cancer *N*=1089	0.299	0.198	0.401
Men with screen-detected cancer *N*=579	0.272	0.172	0.394
Men with clinically detected cancer *N*=510	0.329	0.229	0.408

Polygenic risk score is based on the known 66 prostate cancer susceptibility loci.

a By setting the mean polygenic relative risk in the population equal to unity.

**Table 3:** Comparison of Gleason score, stage, and PSA categories among men in the trial and men with PRS

	**No. of men** ***N*** **(%)**	**No. of men with PRS** ***N*** **(%)**	***P*** **-value (** ***Z*** **-test)**	**Lower risk group** ***N*** **(%)**	**Higher risk group** ***N*** **(%)**	***P*** **value (** ***χ*** ^***2***^ **-square test)**
**Gleason score**						
Overall	*N*=7268	*N*=1083	<0.001	*N*=288	*N*=795	0.004
Gleason score <7	3947 (54)	731 (68)		214 (74)	517 (65)	
Gleason score ⩾7	3321 (46)	352 (32)		74 (26)	278 (35)	
Screen-detected	*N*=1643	*N*=578	0.645	*N*=163	*N*=415	0.005
Gleason score <7	1204 (73)	416 (72)		131 (80)	285 (69)	
Gleason score ⩾7	439 (27)	162 (28)		32 (20)	130 (31)	
Clinically diagnosed	*N*=5625	*N*=505	<0.001	*N*=125	*N*=380	0.284
Gleason score <7	2743 (49)	315 (62)		83 (66)	232 (61)	
Gleason score ⩾7	2882 (51)	190 (38)		42 (34)	148 (39)	
**Stage**						
Overall	*N*=7448	*N*=1089	<0.001	*N*=289	*N*=800	0.198
Localised stage	5970 (80)	973 (89)		264 (91)	709 (89)	
Advanced stage	1448 (20)	116 (11)		25 (9)	91 (11)	
Screen-detected	*N*=1646	*N*=579	0.441	*N*=163	*N*=416	0.046
Localised stage	1508 (92)	538 (93)		157 (96)	381 (92)	
Advanced stage	138 (8)	41 (7)		6 (4)	35 (8)	
Clinically diagnosed	*N*=5772	*N*=510	<0.001	*N*=126	*N*=384	0.891
Localised stage	4462 (77)	435 (85)		107 (85)	328 (85)	
Advanced stage	1310 (23)	75 (15)		19 (15)	56 (15)	
**PSA at screening**						
Screening arm	*N*=23 770	*N*=4605	<0.001	*N*=2142	*N*=2463	<0.001
PSA <4 ng ml^−1^	19 908 (84)	4016 (87)		1989 (93)	2027 (82)	
PSA ⩾4 ng ml^−1^	3862 (16)	589 (13)		153 (7)	436 (18)	

Abbreviations: PRS=polygenic risk score; PSA=prostate specific antigen.

**Table 4:** Summary of the estimates used to derive the proportion of overdiagnosis by polygenic risk groups

	**Overall**	**Lower risk group**	**Higher risk group**	**95% CI**
**Estimates used to derive expected number of screen-detected cancers**				
Sensitivity (*S*)^a^	0.55	0.55	0.55	0.45–0.65
Inverse of mean sojourn time (*λ*)^a^	0.16	0.16	0.16	0.12–0.24
Baseline incidence rate (cases/person-years) (I*)	0.00617	0.00247^b^	0.00990^c^	
**Sampling fractions**				
No cancer (*α*_*p*_)	1.000	0.502	0.498	
Prevalent screen-detected cancer	1.000	0.243	0.757	
Incident screen-detected cancer	1.000	0.298	0.702	
Interval cancer	1.000	0.200	0.800	

a *S* and *λ* were derived from data on men in the lower and higher risk groups combined.

b *R*_l_.

c *R*_h_.

**Table 5:** Proportion of screen-detected cancers which are likely to be overdiagnosed by polygenic risk

**Screening round**	**No. of screening episodes**	**No. of screen-detected cancer**	**Expected no. of non-overdiagnosed screen-detected cancer**	**Per cent** **(%) overdiagnosis (95% CI)**
**Overall**				
Screening round 1	23 771	686	504	
Screening round 2	18 044	596	272	
Screening round 3	10 328	364	173	
Total	52 143	1646	949	42 (37–52)
**PRS risk groups**				
**Lower risk group**				
Screening round 1	11 938	167	101	
Screening round 2	9062	178	55	
Screening round 3	5187	108	35	
Total	26 186	453	191	58 (54–65)
**Higher risk group**				
Screening round 1	11 833	519	402	
Screening round 2	8982	418	217	
Screening round 3	5141	256	139	
Total	25 957	1193	758	37 (31–47)
