# Supplementary material for: Reducing overdiagnosis by polygenic risk-stratified screening: findings from the Finnish section of the ERSPC
Source: Br J Cancer. 2015 Aug 20;113(7):1086–93. doi: 10.1038/bjc.2015.289 (PMC4651137; doi:10.1038/bjc.2015.289)
Supplement: Supplementary Information [file bjc2015289x1.docx]

**Supplementary file:**

**Genotyping and quality control**

We obtained the genotyping data on a sample of the trial participants (1,089 men with prostate cancer and 3,878 men without prostate cancer) in two genotyping phases. The genotyping outcome for 2,851 men on then known 70 prostate cancer susceptibility SNPs were obtained from genotyping performed using a custom Illumina Infinium array (iCOGS), as described previously (Eeles et al.,2013 ). DNA samples from an additional 2,116 men were genotyped for 60 of these SNPs and for 10 highly correlated, surrogate SNPs (r^2^>0.75) using Fluidigm Corporation’s BioMark 96.96 Dynamic Array and TaqMan SNP assays according to the manufacturer’s instructions (Wang et al.,2009 ). Both genotyping rounds were done in the same laboratory. In each 384-well plate, 2% duplicates and 1% PCR-negative controls (with no DNA) were included. Genotype intensity cluster plots were visually inspected and the data excluded if clustering was judged to be poor. We excluded individuals with more than 10% missing genotypes, and excluded variants without genotype call (rs3850699, rs6062509); one variant with a call rate less than 90% (rs675495); and one variant with genotype frequency that deviated from Hardy-Weinberg equilibrium in controls at p<0.005 (rs1465618). For individuals with less than 10% missing genotype, we imputed the expected value based on the observed allele frequency. To harmonise the genotype calls in the two rounds of genotyping, when a variant was excluded, its surrogate marker was excluded too (rs339331 as surrogate SNP to rs675495). Consequently, the analysis was based on data from these 66 SNPs associated at genome wide significance level (p<10^-8^) with susceptibility for prostate cancer.

**References**

Eeles RA, Olama AA, Benlloch S, Saunders EJ, Leongamornlert DA, Tymrakiewicz M, Ghoussaini M, Luccarini C, Dennis J, Jugurnauth-Little S, Dadaev T, Neal DE, Hamdy FC, Donovan JL, Muir K, Giles GG, Severi G, Wiklund F, Gronberg H, Haiman CA, Schumacher F, Henderson BE, Le ML, Lindstrom S, Kraft P, Hunter DJ, Gapstur S, Chanock SJ, Berndt SI, Albanes D, Andriole G, Schleutker J, Weischer M, Canzian F, Riboli E, Key TJ, Travis RC, Campa D, Ingles SA, John EM, Hayes RB, Pharoah PD, Pashayan N, Khaw KT, Stanford JL, Ostrander EA, Signorello LB, Thibodeau SN, Schaid D, Maier C, Vogel W, Kibel AS, Cybulski C, Lubinski J, Cannon-Albright L, Brenner H, Park JY, Kaneva R, Batra J, Spurdle AB, Clements JA, Teixeira MR, Dicks E, Lee A, Dunning AM, Baynes C, Conroy D, Maranian MJ, Ahmed S, Govindasami K, Guy M, Wilkinson RA, Sawyer EJ, Morgan A, Dearnaley DP, Horwich A, Huddart RA, Khoo VS, Parker CC, Van As NJ, Woodhouse CJ, Thompson A, Dudderidge T, Ogden C, Cooper CS, Lophatananon A, Cox A, Southey MC, Hopper JL, English DR, Aly M, Adolfsson J, Xu J, Zheng SL, Yeager M, Kaaks R, Diver WR, Gaudet MM, Stern MC, Corral R, Joshi AD, Shahabi A, Wahlfors T, Tammela TL, Auvinen A, Virtamo J, Klarskov P, Nordestgaard BG, Roder MA, Nielsen SF, Bojesen SE, Siddiq A, FitzGerald LM, Kolb S, Kwon EM, Karyadi DM, Blot WJ, Zheng W, Cai Q, McDonnell SK, Rinckleb AE, Drake B, Colditz G, Wokolorczyk D, Stephenson RA, Teerlink C, Muller H, Rothenbacher D, Sellers TA, Lin HY, Slavov C, Mitev V, Lose F, Srinivasan S, Maia S, Paulo P, Lange E, Cooney KA, Antoniou AC, Vincent D, Bacot F, Tessier DC, Kote-Jarai Z, Easton DF (2013) Identification of 23 new prostate cancer susceptibility loci using the iCOGS custom genotyping array. *Nat Genet* **45** (4): 385-391

Wang J, Lin M, Crenshaw A, Hutchinson A, Hicks B, Yeager M, Berndt S, Huang WY, Hayes RB, Chanock SJ, Jones RC, Ramakrishnan R (2009) High-throughput single nucleotide polymorphism genotyping using nanofluidic Dynamic Arrays. *BMC Genomics* **10** 561

**Supplementary Table 1S**. Common susceptibility loci for prostate cancer included in deriving polygenic risk score

| Locus | SNP | Proxy SNP | r2* | Effect allele | Reference allele | Effect allele frequency† | Per allele OR | 95% CI | | | Ref |  |
| --- | --- | --- | --- | --- | --- | --- | --- | --- | --- | --- | --- | --- |
| 1q21 | rs1218582 |  |  | G | A | 0.45 | 1.06 | 1.03 | 1.09 | | 1 | |
| 1q32 | rs4245739 |  |  | C | A | 0.25 | 0.91 | 0.88 | 0.95 | | 1 | |
| 2p11 | rs10187424 | rs1009 | 0.96 | G | A | 0.41 | 0.92 | 0.89 | 0.94 | | 2 | |
| 2p11 | rs1009 | rs10187424 |  | G | A | 0.43 | 0.91 | 0.94 | 0.89 | |  |  |
| 2p15 | rs721048 |  |  | A | G | 0.19 | 1.13 | 1.09 | 1.16 | | 3 | |
| 2p21 | rs1465618 |  |  | A | G | 0.22 | 1.08 | 1.05 | 1.11 | | 4 | |
| 2p24 | rs13385191 | rs13394027 | 0.89 | G | A | 0.56 | 1.15 | 1.10 | 1.21 | | 5 | |
| 2p24 | rs13394027 | rs13385191 |  | A | G | 0.22 | 1.05 | 1.01 | 1.08 | |  |  |
| 2p25 | rs11902236 |  |  | A | G | 0.27 | 1.07 | 1.03 | 1.10 | | 1 | |
| 2q31 | rs12621278 |  |  | G | A | 0.05 | 0.76 | 0.69 | 0.83 | | 4 | |
| 2q37 | rs2292884 |  |  | G | A | 0.24 | 1.06 | 1.03 | 1.10 | | 2,6 | |
| 2q37 | rs3771570 |  |  | A | G | 0.15 | 1.12 | 1.08 | 1.16 | | 1 | |
| 3p11 | rs2055109 | rs1494248 | 0.78 | C | T | 0.90 | 1.20 | 1.13 | 1.29 | | 7 | |
| 3p11 | rs1494248 | rs2055109 |  | C | A | 0.30 | 0.98 | 0.96 | 1.01 | |  |  |
| 3p12 | rs2660753 |  |  | A | G | 0.11 | 1.13 | 1.08 | 1.17 | | 8 | |
| 3q13 | rs7611694 |  |  | C | A | 0.41 | 0.91 | 0.88 | 0.94 | | 1 | |
| 3q21 | rs10934853 |  |  | A | C | 0.29 | 1.12 | 1.08 | 1.15 | | 9 | |
| 3q23 | rs6763931 |  |  | A | G | 0.45 | 1.03 | 1.01 | 1.06 | | 2 | |
| 3q26 | rs10936632 | rs10804839 | 0.76 | C | A | 0.48 | 0.9 | 0.88 | 0.93 | | 2 | |
| 3q26 | rs10804839 | rs10936632 |  | A | T | 0.45 | 1.09 | 1.06 | 1.12 | |  |  |
| 4q13 | rs1894292 |  |  | A | G | 0.48 | 0.91 | 0.88 | 0.94 | | 1 | |
| 4q22 | rs12500426 |  |  | A | C | 0.48 | 1.09 | 1.06 | 1.12 | | 4 | |
| 4q22 | rs17021918 |  |  | A | G | 0.34 | 0.90 | 0.87 | 0.93 | | 4 | |
| 4q24 | rs7679673 |  |  | A | C | 0.39 | 0.86 | 0.83 | 0.89 | | 4 | |
| 5p12 | rs2121875 | rs1482679 | 1.00 | G | T | 0.34 | 1.05 | 1.02 | 1.08 | | 2 | |
| 5p12 | rs1482679 | rs2121875 |  | G | A | 0.33 | 1.07 | 1.04 | 1.1 | |  |  |
| 5p15 | rs12653946 |  |  | A | G | 0.43 | 1.08 | 1.05 | | 1.11 | 5 | |
| 5q35 | rs6869841 |  |  | A | G | 0.21 | 1.07 | 1.04 | | 1.11 | 1 | |
| 6p21 | rs130067 |  |  | C | A | 0.21 | 1.07 | 1.03 | | 1.10 | 2 | |
| 6p21 | rs1983891 | rs913074 | 1.00 | T | C | 0.41 | 1.15 | 1.09 | | 1.21 | 5 | |
| 6p21 | rs913074 | rs1983891 |  | G | A | 0.28 | 1.07 | 1.04 | | 1.10 |  |  |
| 6p21 | rs2273669 |  |  | G | A | 0.15 | 1.07 | 1.03 | | 1.11 | 1 | |
| 6q25 | rs1933488 |  |  | G | A | 0.41 | 0.89 | 0.86 | | 0.92 | 1 | |
| 6q25 | rs9364554 |  |  | A | G | 0.30 | 1.10 | 1.07 | | 1.13 | 8 | |
| 7p15 | rs10486567 |  |  | A | G | 0.22 | 0.87 | 0.83 | | 0.9 | 10 | |
| 7p21 | rs12155172 |  |  | A | G | 0.23 | 1.11 | 1.07 | | 1.14 | 1 | |
| 7q21 | rs6465657 |  |  | G | A | 0.48 | 1.12 | 1.09 | | 1.15 | 8 | |
| 8p21 | rs11135910 |  |  | A | G | 0.16 | 1.11 | 1.07 | | 1.15 | 1 | |
| 8p21 | rs1512268 |  |  | A | G | 0.45 | 1.13 | 1.10 | | 1.16 | 4 | |
| 8p21 | rs2928679 |  |  | A | G | 0.44 | 1.05 | 1.02 | | 1.08 | 4 | |
| 8q24 | rs10086908 |  |  | G | A | 0.29 | 0.87 | 0.84 | | 0.90 | 11 | |
| 8q24 | rs12543663 |  |  | C | A | 0.31 | 1.15 | 1.12 | | 1.18 | 11 | |
| 8q24 | rs1447295 |  |  | A | C | 0.13 | 1.43 | 1.39 | | 1.48 | 12 | |
| 8q24 | rs16901979 |  |  | A | C | 0.04 | 1.66 | 1.59 | | 1.74 | 13 | |
| 8q24 | rs620861 |  |  | A | G | 0.35 | 0.87 | 0.84 | | 0.90 | 11 | |
| 8q24 | rs6983267 |  |  | A | C | 0.49 | 0.8 | 0.78 | | 0.83 | 14 | |
| 9q31 | rs817826 |  |  | C | T | 0.08 | 1.41 | 1.29 | | 1.54 | 15 | |
| 9q33 | rs1571801 |  |  | A | C | 0.28 | 1.03 | 1 | | 1.07 | 16 | |
| 10q11 | rs10993994 |  |  | A | G | 0.41 | 1.23 | 1.2 | | 1.26 | 8,10 | |
| 10q26 | rs2252004 | rs2252344 | 1.00 | G | T | 0.77 | 1.16 | 1.10 | | 1.22 | 7 | |
| 10q26 | rs2252344 | rs2252004 |  | A | G | 0.10 | 0.98 | 0.94 | | 1.03 |  |  |
| 10q26 | rs4962416 |  |  | G | A | 0.27 | 1.05 | 1.02 | | 1.08 | 10 | |
| 11p15 | rs7127900 |  |  | A | G | 0.21 | 1.24 | 1.21 | | 1.28 | 4 | |
| 11q13 | rs7931342 |  |  | A | C | 0.48 | 0.83 | 0.80 | | 0.86 | 8,10 | |
| 11q22 | rs11568818 |  |  | G | A | 0.44 | 0.91 | 0.88 | | 0.94 | 1 | |
| 12q13 | rs10875943 |  |  | G | A | 0.30 | 1.10 | 1.07 | | 1.13 | 2 | |
| 12q13 | rs902774 |  |  | A | G | 0.16 | 1.13 | 1.09 | | 1.16 | 6 | |
| 12q24 | rs1270884 |  |  | A | G | 0.49 | 1.07 | 1.04 | | 1.10 | 1 | |
| 14q22 | rs8008270 |  |  | A | G | 0.18 | 0.89 | 0.86 | | 0.93 | 1 | |
| 14q24 | rs7141529 |  |  | G | A | 0.49 | 1.09 | 1.06 | | 1.12 | 1 | |
| 17p13 | rs684232 |  |  | G | A | 0.36 | 1.10 | 1.07 | | 1.13 | 1 | |
| 17q12 | rs11649743 |  |  | A | G | 0.19 | 0.88 | 0.84 | | 0.92 | 17 | |
| 17q12 | rs4430796 | rs11651755 | 1.00 | A | G | 0.49 | 1.22 | 1.15 | | 1.3 | 13 | |
| 17q12 | rs11651755 | rs4430796 |  | G | A | 0.48 | 0.81 | 0.79 | | 0.83 |  |  |
| 17q21 | rs11650494 |  |  | A | G | 0.08 | 1.15 | 1.1 | | 1.21 | 1 | |
| 17q24 | rs1859962 |  |  | A | C | 0.52 | 0.84 | 0.81 | | 0.87 | 13 | |
| 18q23 | rs7241993 |  |  | A | G | 0.30 | 0.92 | 0.89 | | 0.95 | 1 | |
| 19q13 | rs103294 |  |  | A | G | 0.22 | 1.00 | 0.97 | | 1.04 | 15 | |
| 19q13 | rs11672691 |  |  | A | G | 0.26 | 0.9 | 0.87 | | 0.93 | 18 | |
| 19q13 | rs2735839 |  |  | A | G | 0.13 | 0.83 | 0.79 | | 0.88 | 8 | |
| 19q13 | rs8102476 |  |  | A | G | 0.46 | 0.93 | 0.9 | | 0.96 | 9 | |
| 20q13 | rs2427345 |  |  | A | G | 0.37 | 0.94 | 0.91 | | 0.97 | 1 | |
| Xp11 | rs5945619 |  |  | G | A | 0.40 | 1.11 | 1.09 | | 1.13 | 3,8 | |
| Xq12 | rs5919432 |  |  | G | A | 0.19 | 0.96 | 0.94 | | 0.99 | 2 | |

* Correlation between two proxy SNPs

†Derived from the genotyping of 211,155 SNPs on a custom Illumina array (iCOGS) in blood from 25,074 prostate cancer cases and 24,272 controls from the international PRACTICAL Consortium^1^

Abbreviations: A, adenine; C, cytosine; G, guanine; T, tyrosine

**References**

1. Eeles RA, Olama AA, Benlloch S et al (2013) Identification of 23 new prostate cancer

susceptibility loci using the iCOGS custom genotyping array. Nat Genet; 45(4):385-391.

2. Kote-Jarai Z, Olama AA, Giles GG et al (2011) Seven prostate cancer susceptibility loci

identified by a multi-stage genome-wide association study. Nat Genet; 43(8):785-791.

3. Gudmundsson J, Sulem P, Rafnar T et al (2008) Common sequence variants on 2p15 and Xp11.22 confer susceptibility to prostate cancer. Nat Genet; 40(3):281-283.

4. Eeles RA, Kote-Jarai Z, Al Olama AA et al (2009) Identification of seven new prostate cancer susceptibility loci through a genome-wide association study. Nat Genet; 41(10):1116-1121.

5. Takata R, Akamatsu S, Kubo M et al (2010) Genome-wide association study identifies five new susceptibility loci for prostate cancer in the Japanese population. Nat Genet; 42(9):751-754.

6. Schumacher FR, Berndt SI et al (2011) Genome-wide association study identifies new prostate cancer susceptibility loci. Hum Mol Genet; 20(19):3867-3875.

7. Akamatsu S, Takata R, Haiman CA et al (2012) Common variants at 11q12, 10q26 and 3p11.2 are associated with prostate cancer susceptibility in Japanese. Nat Genet; 44(4):426-9, S1.

8. Eeles RA, Kote-Jarai Z, Giles GG et al (2008) Multiple newly identified loci associated with prostate cancer susceptibility. Nat Genet; 40(3):316-321.

9. Gudmundsson J, Sulem P, Gudbjartsson DF et al (2009) Genome-wide association and replication studies identify four variants associated with prostate cancer susceptibility. Nat Genet; 41(10):1122-1126.

10. Thomas G, Jacobs KB, Yeager M et al (2008). Multiple loci identified in a genome-wide association study of prostate cancer. Nat Genet; 40(3):310-315.

11. Al Olama AA, Kote-Jarai Z, Giles GG et al (2009) Multiple loci on 8q24 associated with prostate cancer susceptibility. Nat Genet; 41(10):1058-1060.

12. Amundadottir LT, Sulem P, Gudmundsson J et al (2006) A common variant associated with prostate cancer in European and African populations. Nat Genet; 38(6):652-658.

13. Gudmundsson J, Sulem P, Manolescu A et al (2007) Genome-wide association study identifies a second prostate cancer susceptibility variant at 8q24. Nat Genet; 39(5):631-637.

14. Yeager M, Orr N, Hayes RB et al (2007) Genome-wide association study of prostate cancer identifies a second risk locus at 8q24. Nat Genet; 39(5):645-649.

15. Xu J, Mo Z, Ye D et al (2012) Genome-wide association study in Chinese men identifies two new prostate cancer risk loci at 9q31.2 and 19q13.4. Nat Genet; 44(11):1231-1235.

16. Duggan D, Zheng SL, Knowlton M et al (2007) Two genome-wide association studies of aggressive prostate cancer implicate putative prostate tumor suppressor gene DAB2IP. J Natl Cancer Inst; 99(24):1836-1844.

17. Sun J, Zheng SL, Wiklund F et al (2008) Evidence for two independent prostate cancer risk-associated loci in the HNF1B gene at 17q12. Nat Genet; 40(10):1153-1155.

18. Al Olama A, Kote-Jarai Z, Schumacher FR et al (2013) A meta-analysis of genome-wide association studies to identify prostate cancer susceptibility loci associated with aggressive and non-aggressive disease. Hum Mol Genet; 22(2):408-415.
